# Exploring the Association of Leukocyte Telomere Length and Hearing Threshold Shifts of Adults in the United States

**DOI:** 10.3389/fnagi.2022.770159

**Published:** 2022-06-03

**Authors:** Lili Long, Zhaoli Meng, Zhenchao Jia, Xinghua Tang

**Affiliations:** ^1^Department of Otorhinolaryngology, Sichuan University Hospital of Sichuan University, Chengdu, China; ^2^Department of Otorhinolaryngology-Head and Neck Surgery, West China Hospital of Sichuan University, Chengdu, China; ^3^Department of Prevention and Health Care, Sichuan University Hospital of Sichuan University, Chengdu, China; ^4^Department of Otorhinolaryngology-Head and Neck Surgery, Sichuan Provincial People’s Hospital, University of Electronic Science and Technology of China, Chengdu, China

**Keywords:** telomere length, hearing threshold shift, National Health and Nutrition Examination Survey, cross-sectional study, adults

## Abstract

**Background:**

Although telomere length has a significant relationship with various age-related diseases, studies on its relationship with hearing status in adults are limited and equivocal. This study investigated the associations between mean telomere length (MTL) and low-, speech-, and high-frequency hearing threshold shifts of adults in the United States.

**Methods:**

A total of 2,027 adults, aged 20-69 years, from the National Health and Nutrition Examination Surveys (NHANES, 1999–2002) were included in the analytic sample. The quantitative polymerase chain reaction method was used for the MTL assay, and MTL was expressed using the telomere-to-single copy gene (T/S) ratio. Hearing loss was defined as a pure-tone average (PTA) for the better ear at ≥ 20 dB HL at frequencies 500, 1,000, 2,000, and 4,000 Hz. Univariate and multivariate linear regression analyses and smooth curve fittings were conducted to evaluate the correlation between MTL and low-, speech-, and high-frequency hearing levels.

**Results:**

The mean age of the participants was 40.60 ± 12.76 years, including 952 men (weighted, 48.67%) and 303 (weighted, 12.88%) participants with hearing loss. After adjusting for potential confounders in the multivariate linear regression model, the relationship between MTL and hearing thresholds was not statistically significant. Smooth curve fittings indicated a non-linear relationship between MTL and high-frequency PTA hearing threshold shifts. MTL was inversely related to high-frequency PTA to the turning point (T/S ratio = 0.82) (adjusted β−21.45, 95% CI −37.28, −5.62; *P* = 0.008). When the T/S ratio exceeded0.82, MTL was not associated with high-frequency PTA (adjusted β0.18, 95% CI −2.21, 2.57; *P* = 0.8809).

**Conclusion:**

Our findings revealed that MTL was associated with high-frequency PTA hearing threshold shifts of adults in the United States in a non-linear manner.

## Introduction

Hearing loss, which is associated with lifelong adverse health consequences and affects social and economic development, is the most common sensory disability in humans (Bowl et al., 2017; Cunningham and Tucci, 2017). It can originate in childhood (Vos et al., 2017) and is increasingly prevalent in adults (Agrawal et al., 2008). Genetic and environmental factors can cause hearing loss. Although genetic mutations, noise exposure, ototoxic drugs, and aging are the most studied risk factors for hearing loss, understanding of the underlying biological and molecular mechanisms of hearing loss is still limited.

Telomeres are repetitive DNA sequences (TTAGGG in vertebrates) and associated proteins at the ends of chromosomes for chromosomal stability and cellular integrity (Blackburn et al., 2015). They are considered biomarkers of aging because they shorten with every cell division (Chakravarti et al., 2021). Many age-associated diseases and disorders, such as hypertension, cognitive performance disorder, and cancer mortality, have been studied (Huang et al., 2020; Linghui et al., 2020; Shen et al., 2020). Oxidative stress and inflammation, which can promote hearing loss, are associated with telomere loss (von Zglinicki et al., 2001; Chester et al., 2021; Heba et al., 2021). Thus, telomere length may predispose hearing status and telomere loss may be associated with the biological mechanisms of hearing loss.

To date, the results of studies on the relationship between telomere length and hearing level are quite limited and inconsistent (Liu H. et al., 2017; Wang et al., 2019; Zhang et al., 2020). One study showed that longer telomere length was negatively related to hearing loss in older adults (Liu H. et al., 2017). In another study, telomere length was considered a predictive biomarker of hearing loss at an early stage (Zhang et al., 2020). However, telomere length was not correlated with hearing levels in children and midlife adults in a third study (Wang et al., 2019). Therefore, this study investigated the relationship between mean telomere length (MTL) and hearing level of adults in the United States using the National Health and Nutrition Examination Survey (NHANES) database.

## Materials and Methods

### Ethics Statement

Our study acquired publicly accessible data from the NHANES website^1^. The NHANES data were approved by the National Center for Health Statistics Institutional Review Board by the revised Declaration of Helsinki. Data collection procedures and examinations were performed after informed consent was obtained from all the eligible participants.

### Study Population

**FIGURE 1:**
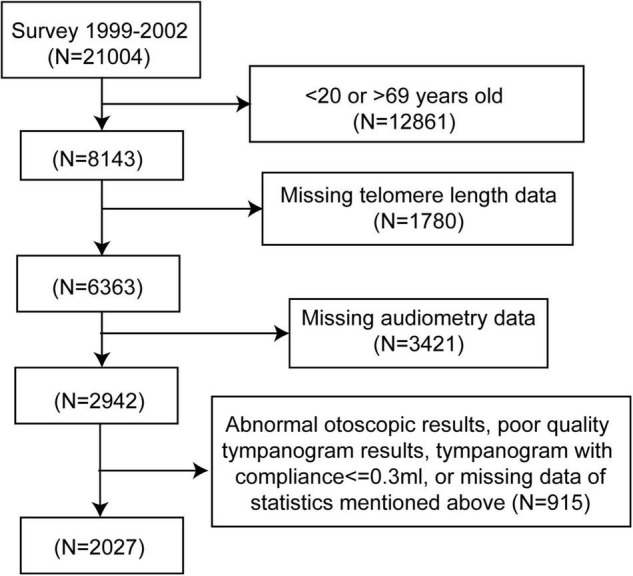
Flow chart of the selection process. NHANES, National Health and Nutrition Examination Survey.

The NHANES is a nationally representative survey approved by the Institutional Review Board of the National Center for Health Statistics. The survey combines a series of interviews, physical examinations, and laboratory tests to collect health-related information from the general population in the United States. The population in this study was enrolled in two cycles of NHANES (1999—2000, 2001–2002), as these are the only cycles containing results of the leukocyte telomere length test. A flow chart for the selection of study participants is shown in Figure 1. Audiometry examinations were performed in adults aged 20–69 years. Participants without complete data on hearing levels, otoscopic test, tympanogram test, or leukocyte telomere length measurement were excluded, as well as participants with abnormal otoscopic results, poor-quality tympanogram results, or tympanogram with compliance of ≤ 0.3ml. Finally, 2,027 adults were included in the study.

### Leukocyte Telomere Length Measurement

DNA from the participants was purified from whole blood and stored at −80°C using standardized procedures before the MTL assay measurement. The MTL assay was performed in the laboratory at the University of California, San Francisco, using the quantitative polymerase chain reaction-based method to measure telomere length relative to standard reference DNA (telomere-to-single copy gene [T/S] ratio), as previously described in detail (Cawthon, 2002; Lin et al., 2010; Needham et al., 2013). More details regarding the MTL quantification procedure and analytical methods are available on the NHANES website^2^.

### Audiometric Measurement

Standardized air conduction pure-tone audiometric measurements were conducted in a sound-isolated room by trained and certificated audiologists. Air-conduction thresholds were tested in both ears of the participants at frequencies between 500 and 8,000 Hz, each with an intensity range of −10 to 120 dB. The hearing threshold was defined as the level at which participants were able to detect 50% of the signal. The 1,000 Hz frequency was tested twice in each ear to ensure quality and reliability (Su and Chan, 2017). Pure-tone average (PTA) hearing thresholds were regarded as: 0.5, 1, and 2 kHz low-frequency; 0.5, 1, 2, and 4 kHz speech-frequency; 4, 6, and 8 kHz high-frequency. Hearing impairment was defined as a hearing threshold of 20 dB or greater at speech-frequency PTA in the better ear (World Health Organization [WHO], 2021).

### Covariates

Potential covariates considered in the analyses included age, body mass index (BMI), sex, race/ethnicity, education level, diabetes, hypertension, cigarette smoking, and noise exposure. Information on age, sex, race/ethnicity, education level, diabetes, and hypertension were obtained during the in-home interviews. Information on noise exposure was obtained from a pre-exam audiometric questionnaire. BMI data were recorded during the physical examination.

Diabetes was determined if the participants answered responded ‘yes’ to “other than during pregnancy, ever been told by a doctor or health professional had diabetes or sugar diabetes.” The answer of “borderline” was also considered diabetes (Szeto et al., 2021). Hypertension was determined by a positive reply to “ever been told by a doctor or other health professional had hypertension, also called high blood pressure” (Szeto et al., 2021). Smoking status was categorized as “ever” or “never” from the answers to the questions, “Have you smoked at least 100 cigarettes in your entire life?” and “Do you now smoke cigarettes?” (Szeto et al., 2021). Noise exposure was defined as “exposed to loud noise or listening to music with headphones in the past 24 h” (Ding and Park, 2020).

### Statistical Analysis

**FIGURE 2:**
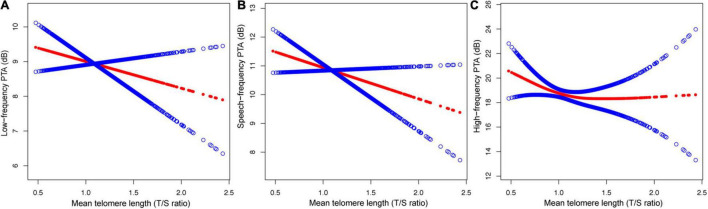
The relationship between MTL and hearing threshold shifts: **(A)** low-frequency PTA, **(B)** speech-frequency PTA and **(C)** high-frequency PTA.

The 1999/2000 and 2001/2002 cycles were combined, and audiometry subsample of 4-year Mobile Examination Center weights (WTSAU4YR) of the two cycles were used to estimate more representative measures for the general population of the United States following the NHANES analytic guidelines (Zipf et al., 2013). Categorical data are shown as percentages and continuous data are presented as means ± standard deviation (SD) according to baseline MTL in quartiles (Table 1). The *P-value* of continuous data was calculated by the weighted linear regression model and the *P-value* of categorical data was calculated by the weighted chi-square test. A univariate analysis was conducted to estimate potential variables (Table 2). A multivariate linear regression analysis was used to determine regression coefficients (β) and 95% confidence intervals (CIs) between MTL and low-, speech-, and high-frequency PTAs (Table 3). Three regression models were built to adjust for the relevant covariates. In the crude mode, no adjustments were included. In the model I, we adjusted for sex and age. In model II, sex, age, race, education level, BMI, noise exposure, hypertension, diabetes, and cigarette smoking status were adjusted. The βs and 95% CIs of low-, speech-, and high-frequency PTAs across each MTL/age, MTL/sex, and MTL/race subgroup were analyzed and their interactions were estimated. Smooth curve fittings were used to explore the relationship between MTL and hearing threshold shifts, with an adjustment for potential confounders (Figure 2). A two piecewise linear regression model was then conducted to examine the threshold effect of MTL on hearing threshold shifts in terms of the smoothing plot. The log-likelihood ratio test was performed to determine whether a threshold existed. The inflection point was calculated using the recursive method, and the maximum model likelihood was used. Statistical significance was set at *P* < 0.05. Statistical analyses were performed using the R statistical programming language 3.6.1 (R Foundation for Statistical Computing), and EmpowerStats software (X&Y Solutions, Inc.).

**TABLE 1:** The weighted demographic characteristics of study participants.

Variables	Total	Quantile of MTL				*P* value
		Q1 (0.471-0.892) (*n* = 507)	Q2 (0.892-1.047) (*n* = 506)	Q3 (1.047-1.232) (*n* = 507)	Q4 (1.232-2.429) (*n* = 507)	
**Continuous variables, mean ± SD**						
Age (year)	40.60 ± 12.76	46.25 ± 12.88	42.52 ± 12.14	38.45 ± 12.21	35.91 ± 11.43	< 0.0001
BMI (kg/m^2^)	28.09 ± 6.42	28.86 ± 6.87	28.51 ± 6.54	27.67 ± 6.13	27.43 ± 6.08	0.0009
Low-frequency PTA	8.44 ± 7.30	9.89 ± 8.04	9.31 ± 7.92	8.17 ± 6.87	6.61 ± 5.84	< 0.0001
Speech-frequency PTA	10.34 ± 8.70	12.57 ± 9.64	11.46 ± 9.17	9.70 ± 8.24	7.95 ± 6.98	< 0.0001
High-frequency PTA	18.25 ± 16.68	23.00 ± 19.13	20.11 ± 16.92	16.45 ± 15.79	14.07 ± 13.37	< 0.0001
**Categorical variables,%**						
Sex (Female)	51.33	50.48	51.36	50.43	52.92	0.8388
Race/Ethnicity						< 0.0001
Mexican American	7.50	7.92	6.75	8.70	6.66	
Non-Hispanic White	71.19	70.07	76.11	74.22	64.54	
Non-Hispanic Black	9.11	7.65	7.78	8.15	12.58	
Other races	12.21	14.36	9.36	8.94	16.21	
Education level						0.1910
Below high school	18.07	19.95	19.44	16.74	16.41	
High school	25.34	27.02	26.09	26.07	22.45	
Above high school	56.59	53.03	54.47	57.19	61.14	
Noise exposure	8.55	3.58	9.39	10.71	10.01	0.0002
BMI (kg/m^2^)						0.0191
Underweight (< 18.5)	1.55	1.39	2.09	1.14	1.57	
Normal (≥ 18.5, < 25)	33.55	26.25	32.91	36.19	37.98	
Overweight (≥ 25, < 30)	34.22	36.93	32.60	34.69	32.92	
Obesity (≥ 30)	29.98	34.48	32.06	26.98	26.98	
Not recorded	0.71	0.94	0.35	1.02	0.56	
Hypertension	19.59	24.28	22.33	16.83	15.58	0.0038
Diabetes	5.94	9.80	5.45	4.82	4.12	0.0029
Cigarette smoking						< 0.0001
Never smoker	50.16	46.98	42.15	55.45	55.39	
Former smoker	23.89	27.92	31.28	18.11	18.99	
Current smoker	25.82	25.11	26.41	26.17	25.53	
Not recorded	0.13		0.16	0.27	0.09	
Hearing loss	12.88	19.16	17.02	10.34	5.92	< 0.0001

*MTL, mean telomere length; SD, standard deviation; BMI, Body Mass Index; PTA, pure tone average.*

**TABLE 2:** The univariate analysis of comparison of variables in hearing threshold group.

Variables	N (%)/Mean ± SD	Low-frequency PTA	Speech-frequency PTA	High-frequency PTA
		β/OR (95% CI) *p* value	β/OR (95% CI) *p* value	β/OR (95% CI) *p* value
**Sex (Female)**	1075 (53.03%)	−0.97 (−1.61, −0.34) 0.0027	−3.54 (−4.28, −2.79) < 0.0001	−8.91 (−10.31, −7.51) < 0.0001
**Age**	41.24 ± 14.01	0.25 (0.23, 0.27) < 0.0001	0.37 (0.34, 0.39) < 0.0001	0.77 (0.72, 0.82) < 0.0001
**Race/Ethnicity**				
Mexican American	494 (24.37%)	Reference	Reference	Reference
Non-Hispanic White	986 (48.64%)	0.57 (−0.65, 1.79) 0.3570	1.18 (−0.27, 2.63) 0.1116	4.08 (1.31, 6.85) 0.0040
Non-Hispanic Black	344 (16.97%)	−0.16 (−1.72, 1.41) 0.8442	−0.41 (−2.27, 1.46) 0.6695	−1.17 (−4.73, 2.39) 0.5196
Other races	203 (10.01%)	1.59 (0.12, 3.06) 0.0347	1.39 (−0.36, 3.15) 0.1200	0.96 (−2.39, 4.31) 0.5758
**Education level**				
Below high school	579 (28.56%)	Reference	Reference	Reference
High school	459 (22.64%)	−2.59 (−3.56, −1.62) < 0.0001	−2.78 (−3.93, −1.62) < 0.0001	−2.58 (−4.81, −0.35) 0.0235
Above high school	989 (48.79%)	−3.15 (−3.99, −2.30) < 0.0001	−3.55 (−4.56, −2.54) < 0.0001	−4.18 (−6.13, −2.22) < 0.0001
**BMI**				
Underweight (< 18.5)	25 (1.23%)	Reference	Reference	Reference
Normal (≥ 18.5, < 25)	615 (30.34%)	0.73 (−1.84, 3.31) 0.5777	1.73 (−1.34, 4.80) 0.2688	4.70 (−1.20, 10.61) 0.1188
Overweight (≥ 25, < 30)	724 (35.72%)	2.63 (0.06, 5.21) 0.0452	4.55 (1.48, 7.62) 0.0037	9.96 (4.05, 15.87) 0.0010
Obesity (≥ 30)	643 (31.72%)	3.59 (1.01, 6.17) 0.0065	4.84 (1.76, 7.92) 0.0021	8.57 (2.64, 14.49) 0.0046
Not recorded	20 (0.99%)	7.15 (2.67, 11.64) 0.0018	9.92 (4.58, 15.27) 0.0003	16.55 (6.26, 26.85) 0.0016
**Noise exposure**	170 (8.39%)	0.52 (−0.62, 1.65) 0.3738	0.49 (−0.87, 1.84) 0.4826	−0.66 (−3.26, 1.94) 0.6185
**Hypertension**	438 (21.61%)	3.22 (2.43, 4.01) < 0.0001	4.15 (3.21, 5.09) < 0.0001	7.23 (5.43, 9.04) < 0.0001
**Diabetes**	148 (7.30%)	4.95 (3.62, 6.27) < 0.0001	6.34 (4.76, 7.92) < 0.0001	12.11 (9.08, 15.14) < 0.0001
**Cigarette smoking**				
Never smoker	1067 (52.64%)	Reference	Reference	Reference
Former smoker	474 (23.38%)	2.43 (1.64, 3.21) < 0.0001	4.17 (3.25, 5.10) < 0.0001	8.78 (7.01, 10.55) < 0.0001
Current smoker	483 (23.83%)	1.83 (1.06, 2.59) < 0.0001	2.17 (1.27, 3.07) < 0.0001	3.01 (1.29, 4.74) 0.0006
Not recorded	3 (0.15%)	3.38 (−5.24, 12.01) 0.4420	4.39 (−5.81, 14.58) 0.3992	4.20 (−15.28, 23.69) 0.6725
**MTL (per 1T/S increment)**	1.08 ± 0.27	−5.01 (−6.19, −3.84) < 0.0001	−6.90 (−8.29, −5.51) < 0.0001	−12.99 (−15.66, −10.31) < 0.0001
**MTL (quartiles)**				
Q1 (0.471-0.892)	507 (25.01%)	Reference	Reference	Reference
Q2 (0.891-1.047)	506 (24.96%)	−0.58 (−1.48, 0.33) 0.2114	−1.10 (−2.17, −0.03) 0.0445	−2.89 (−4.95, −0.84) 0.0059
Q3 (1.047-1.232)	507 (25.01%)	−1.72 (−2.62, −0.82) 0.0002	−2.87 (−3.94, −1.80) < 0.0001	−6.55 (−8.59, −4.50) < 0.0001
Q4 (1.232-2.429)	507 (25.01%)	−3.28 (−4.17, −2.39) < 0.0001	−4.61 (−5.67, −3.55) < 0.0001	−8.93 (−10.96, −6.90) < 0.0001

*SD, standard deviation; PTA, pure tone average; OR, odds ratio; CI, confidence interval; BMI, body mass index; MTL, mean telomere length.*

**TABLE 3:** Multivariable linear regression models for outcome of hearing thresholds.

MTL	β (95% CI), *P* value of PTA levels, dB		
	Crude Model	Model I	Model II
**Low-frequency PTA**			
MTL (per 1T/S increment)	−5.01 (−6.19, −3.84) < 0.0001	−1.56 (−2.68, −0.43) 0.0067	−1.12 (−2.22, −0.01) 0.0474
Q1	Reference	Reference	Reference
Q2	−0.58 (−1.48, 0.33) 0.2114	0.33 (−0.50, 1.16) 0.4303	0.46 (−0.35, 1.27) 0.2656
Q3	−1.72 (−2.62, −0.82) 0.0002	0.17 (−0.67, 1.01) 0.6936	0.46 (−0.36, 1.28) 0.2695
Q4	−3.28 (−4.17, −2.39) < 0.0001	−0.76 (−1.61, 0.09) 0.0808	−0.42 (−1.25, 0.41) 0.3176
*P* for trend	< 0.0001	0.0616	0.2938
**Speech-frequency PTA**			
MTL (per 1T/S increment)	−6.90 (−8.29, −5.51) < 0.0001	−1.76 (−2.98, −0.54) 0.0048	−1.20 (−2.40, 0.00) 0.0503
Q1	Reference	Reference	Reference
Q2	−1.10 (−2.17, −0.03) 0.0445	0.26 (−0.64, 1.17) 0.5666	0.33 (−0.55, 1.21) 0.4629
Q3	−2.87 (−3.94, −1.80) < 0.0001	−0.08 (−1.00, 0.83) 0.8572	0.20 (−0.70, 1.09) 0.6657
Q4	−4.61 (−5.67, −3.55) < 0.0001	−0.83 (−1.76, 0.09) 0.0783	−0.43 (−1.33, 0.47) 0.3501
*P* for trend	< 0.0001	0.0488	0.2962
**High-frequency PTA**			
MTL (per 1T/S increment)	−12.99 (−15.66, −10.31) < 0.0001	−2.07 (−4.26, 0.13) 0.0648	−1.09 (−3.27, 1.09) 0.3278
Q1	Reference	Reference	Reference
Q2	−2.89 (−4.95, −0.84) 0.0059	0.00 (−1.62, 1.62) 0.9983	−0.05 (−1.65, 1.55) 0.9494
Q3	−6.55 (−8.59, −4.50) < 0.0001	−0.66 (−2.30, 0.97) 0.4276	−0.46 (−2.08, 1.16) 0.5771
Q4	−8.93 (−10.96, −6.90) < 0.0001	−0.91 (−2.57, 0.74) 0.2806	−0.25 (−1.89, 1.39) 0.7637
*P* for trend	< 0.0001	0.2008	0.6687

*Crude Model = unadjusted. Model I = Crude Model + sex, age. Model II = Model I + race, education level, BMI, noise exposure, hypertension, diabetes, cigarette smoking. MTL, mean telomere length; CI, confidence interval; PTA, pure tone average.*

## Results

### Characteristics of Participants

The baseline characteristics of the 2,027 participants (weighted mean, aged 40.60 ± 12.76 years) enrolled in this study were stratified by quartiles for MTL and are shown in Table 1. This study included 952 men (weighted, 48.67%) and 1,075 women (weighted, 51.33%). Of the participants, 1,367 (weighted, 64.2%) were above normal weight, 438 (weighted, 19.59%) with hypertension, and 148 (weighted, 5.94%) with diabetes. The means ± SD of low-frequency, speech-frequency, and high-frequency PTA hearing thresholds were 8.44 ± 7.30, 10.34 ± 8.70, and 18.25 ± 16.68 dB, respectively. There were 303 (weighted, 12.88%) participants with hearing loss. In addition, there were significant subgroup differences in age, low-, speech, and high-frequency PTAs, BMI, race/ethnicity, noise exposure, hypertension, diabetes, smoking status, and hearing loss rate (all *P* < 0.01).

### Relationship Between MTL and Hearing Threshold Shifts

In a univariate analysis, sex, age, education level, BMI, hypertension, diabetes, cigarette smoking, and MTL (both continuous MTL and MTL in quartiles) were significantly associated with low-, speech, and high-frequency PTA hearing thresholds (all *P* < 0.01) (Table 2). To further explore the association between MTL and hearing threshold shifts, a multivariate regression analysis was conducted. As shown in Table 3, when treating MTL as a continuous variable (per 1 T/S increment) in the non-adjusted model (crude model), MTL was significantly associated with low-frequency (β = −5.01, 95% CI: −6.19, −3.84; *P* < 0.01), speech-frequency (β = −6.90, 95% CI: −8.29, −5.51; *P* < *0.01*), and high-frequency (β = −12.99, 95% CI: −15.66, −10.31; *P* < *0.01*) PTA hearing threshold shifts. However, in model II, there was no association between MTL and low-frequency (β = −1.12, 95% CI: −2.22, −0.01; *P* = 0.0474), speech-frequency (β = −1.20, 95% CI: −2.40, −0.00; *P* = 0.0503), and high-frequency (β = −1.09, 95% CI: −3.27, −1.09; *P* = 0.3278) PTA hearing threshold shifts. When the lowest quartiles of MTL was the referent, multivariate linear (low-, speech-, high-frequency PTAs) regression analyses demonstrated the βs for low-frequency PTA (βs0.46, 0.46, and −0.42 from the second to the fourth quartiles, respectively; *P* = 0.2938 for trend), speech-frequency PTA (βs0.33, 0.20, and −0.43, respectively, from the second to the fourth quartiles; *P* = 0.2962 for trend) and high-frequency PTA (βs −0.05, −0.46, and −0.25, from the second to the fourth quartiles, respectively; *P* = 0.6687 for trend) in the fully adjusted model (model II). MTL showed no statistically significant relationship with hearing threshold shifts after stratification by either age, sex, or race (Supplementary Tables 1–3).

### Analyses of a Non-linear Relationship Between MTL and Hearing Threshold Shifts

After adjusting for potential confounders, including sex, age, race, education level, BMI, noise exposure, hypertension, diabetes, and cigarette smoking, a non-linear relationship between MTL and high-frequency PTA but not low-, or speech-frequency PTAs was observed (Figure 2). The inflection point was calculated using a two-piecewise linear regression model in the T/S ratio for the association with low-, speech-, and high-frequency PTA hearing threshold shifts to 1.14, 1.06, and 0.82, respectively. On the left of the inflection point, the βs for low-, speech-, and high frequency PTAs were −0.79 (95% CI: −1.45, 3.02; *P* = 0.4913), 0.26 (95% CI: −2.73, 3.25; *P* = 0.8661), and −21.45 (95% CI: −37.28, −5.62; *P* = 0.0080), respectively. The corresponding effect estimates at the right of inflection point were −2.56 (95% CI: −4.41, −0.72; *P* = 0.0065), −1.88 (95% CI: −3.64, −0.13; *P* = −0.0358), and0.18 (95% CI: −2.21, 2.57; *P* = 0.8809), respectively. The high-frequency PTA decreased with the T/S ratio up to the turning point (0.82). When the T/S ratio exceeded0.82, MTL was not associated with the high-frequency PTA (Table 4).

**TABLE 4:** The results of two-piecewise linear regression model between MTL and hearing thresholds.

Exposure variables	Low-frequency PTA	Speech-frequency PTA	High-frequency PTA
Cut off point of MTL	1.14	1.06	0.82
< Cut off point of MTL	0.79 (−1.45, 3.02), 0.4913	0.26 (−2.73, 3.25), 0.8661	−21.45 (−37.28, −5.62), 0.0080
≥ Cut off point of MTL	−2.56 (−4.41, −0.72), 0.0065	−1.88 (−3.64, −0.13), 0.0358	0.18 (−2.21, 2.57), 0.8809
Loglikelihood ratio test	0.054	0.294	0.011

## Discussion

The present nationwide cross-sectional study identified a relationship between telomere length and hearing threshold shifts of adults residing in the United States and indicated that MTL was inversely associated with the high-frequency PTA in a non-linear manner after adjusting for sex, age, race, education level, BMI, noise exposure, hypertension, diabetes, and cigarette smoking (Figure 2 and Table 4). To the best of our knowledge, it is the first cross-sectional study on the relationship between telomere length and hearing threshold shifts of adults in the United States. The findings of this study suggest that telomere length might be a potential predictive biomarker of hearing threshold shifts.

Mammalian telomeres are composed of tandem repeats of the hexanucleotide sequence TTAGGG and several DNA-binding proteins (Blackburn et al., 2015). Telomeres shorten during every cell division with the shortening rate varying among the species, suggesting that they are determinants of species’ life spans and have hallmarks of aging (Whittemore et al., 2019; Chakravarti et al., 2021). A large number of studies have been conducted to prove the correlation between MTL and age-related chronic diseases or disorders, such as cardiovascular disease, cognitive performance, and cancer mortality (Huang et al., 2020; Linghui et al., 2020; Shen et al., 2020; Hoffmann et al., 2021). However, studies on the relationship between MTL and hearing loss, which is a very common age-related chronic disorder, are quite limited, with results of inverse or negative associations between them (Liu H. et al., 2017; Wang et al., 2019; Zhang et al., 2020). Our results are rather consistent with the results of two previous case-control studies of the Chinese population (Liu X. et al., 2017; Zhang et al., 2020). However, no sex difference was observed in the role of telomere shortening in hearing loss in our study, which is inconsistent with a previous report (Zhang et al., 2020). Study designs and methods may be one of the reasons for the inconsistent results among different studies. Multiple factors, such as racial heterogeneity and environmental factors, different methods of auditory and MTL measurements, and different sample sizes should be considered (Aubert and Lansdorp, 2008; Ishikawa et al., 2016; Wang et al., 2019).

The MTL was inversely related to high-frequency PTA before a turning point (T/S ratio = −0.82). The possible mechanisms are as follows: first, telomere length is inversely associated with aging, and high-frequency PTA increases with age; and second, telomere length is closely related to inflammation, oxidative stress, and inhibition of DNA repair, which play an important role in the development of age-related hearing loss (Rizvi et al., 2014; Zhang et al., 2016). Oxidation damage, including cochlear DNA damage caused by reactive oxygen species (ROS), plays a causal role in the development of hearing loss (Someya et al., 2009). Due to the enrichment of the GGG triplet, telomeres are also highly sensitive to damage by ROS (Houben et al., 2008). Further studies among longitudinal cohorts across different life stages and measurement of MTL in the ear tissue are necessary to confirm these findings. Further experimental studies are needed to explore the mechanisms underlying these findings.

Our study has several strengths. The data in this study were obtained from a large and nationally representative sample from the NHANES, which were standardized and reliable. Participants with abnormal results of the otoscopic examination and poor-quality results in tympanogram or tympanogram compliance of ≤0.3 ml were excluded to avoid analyzing data for conductive or mixed hearing loss.

Despite these strengths, the limitations of this study should be considered. The results of this study were not validated because the NHANES is a cross-sectional study. The data representing noise exposure was from a pre-exam audiometric questionnaire involving noise exposure 24 h before the audiometric examination, which may not have reflected the accurate noise exposure status of participants. Some potential confounders were not calculated in the models.

## Conclusion

According to the results of the NHANES data analyses, MTL was associated with high-frequency PTA hearing threshold shifts of adults in the United States in a non-linear manner. MTL might be a potential predictive biomarker for hearing loss.

## Data Availability Statement

The original contributions presented in the study are included in the article/Supplementary Material, further inquiries can be directed to the corresponding author/s.

## Ethics Statement

The studies involving human participants were reviewed and approved by National Center for Health Statistics Institutional Review Board. The patients/participants provided their written informed consent to participate in this study.

## Author Contributions

LL made formal analysis of data and wrote the original draft. ZM completed the methodology. ZJ and XT completed the conceptualization, review and editing, revising, and final approval, and are accountable for all aspects. All authors have approved the final manuscript as submitted.

## Conflict of Interest

The authors declare that the research was conducted in the absence of any commercial or financial relationships that could be construed as a potential conflict of interest. The reviewer DL declared a shared affiliation with the author ZM to the handling editor at the time of review.

## Publisher’s Note

All claims expressed in this article are solely those of the authors and do not necessarily represent those of their affiliated organizations, or those of the publisher, the editors and the reviewers. Any product that may be evaluated in this article, or claim that may be made by its manufacturer, is not guaranteed or endorsed by the publisher.

## Footnotes

1
https://www.cdc.gov/nchs/nhanes/Index.htm

2
https://wwwn.cdc.gov/nchs/nhanes/1999-2000/telo_a.htm
