# Supplementary material for: Exploring the Association of Leukocyte Telomere Length and Hearing Threshold Shifts of Adults in the United States
Source: Front Aging Neurosci. 2022 Jun 3;14:770159. doi: 10.3389/fnagi.2022.770159 (PMC9204082; doi:10.3389/fnagi.2022.770159)
Supplement: Supplementary file 1 [file Table_1.docx]

**Table S1** Adjusted^a^ associations between MTL (T/S ratio) and PTA hearing thresholds stratified by age (N=2027)

| Age, years | N | Low-frequency PTA | Speech-frequency PTA | High-frequency PTA |
| --- | --- | --- | --- | --- |
|  |  | β (95% CI), *P* value of PTA levels, dB | | |
| ≥20, <30 | 514 | -0.50 (-1.96, 0.96), 0.5040 | 0.06 (-1.40, 1.51), 0.9386 | 1.66 (-0.73, 4.05), 0.1736 |
| ≥30, <40 | 491 | -0.22 (-2.42, 1.98), 0.8448 | -0.08 (-2.41, 2.25), 0.9470 | -0.35 (-3.95, 3.25), 0.8499 |
| ≥40, <50 | 414 | -1.48 (-3.66, 0.70), 0.1843 | -1.47 (-4.01, 1.06), 0.2546 | -1.03 (-6.21, 4.15), 0.6978 |
| ≥50, <69 | 608 | -2.21 (-5.05, 0.64), 0.1291 | -3.08 (-6.14, -0.03), 0.0484 | -3.75 (-9.09, 1.59), 0.1690 |
| *P*_interaction_ |  | 0.4499 | 0.0965 | 0.0848 |

^a^ Adjusted for age, sex, race, education level, BMI, hypertension, diabetes, cigarette smoking, noise exposure.
