# Supplementary material for: Exploring the Association of Leukocyte Telomere Length and Hearing Threshold Shifts of Adults in the United States
Source: Front Aging Neurosci. 2022 Jun 3;14:770159. doi: 10.3389/fnagi.2022.770159 (PMC9204082; doi:10.3389/fnagi.2022.770159)
Supplement: Supplementary file 2 [file Table_2.docx]

**Table S2** Adjusted^a^ associations between MTL (T/S ratio) and PTA hearing thresholds stratified by sex (N=2027).

| Sex | N | Low-frequency PTA | Speech-frequency PTA | High-frequency PTA |
| --- | --- | --- | --- | --- |
|  |  | β (95% CI), *P* value of PTA levels, dB | | |
| Male | 952 | -0.94 (-2.47, 0.60), 0.2317 | -1.00 (-2.79, 0.79), 0.2752 | -1.22 (-4.92, 2.49), 0.5197 |
| Female | 1075 | -1.46 (-3.04, 0.13), 0.0726 | -1.43 (-3.02, 0.15), 0.0760 | -0.68 (-2.94, 1.58), 0.5541 |
| *P*_interaction_ |  | 0.9525 | 0.1124 | 0.0005 |

^a^ Adjusted for age, race, education level, BMI, hypertension, diabetes, cigarette smoking, noise exposure.
