# Supplementary material for: Exploring the Association of Leukocyte Telomere Length and Hearing Threshold Shifts of Adults in the United States
Source: Front Aging Neurosci. 2022 Jun 3;14:770159. doi: 10.3389/fnagi.2022.770159 (PMC9204082; doi:10.3389/fnagi.2022.770159)
Supplement: Supplementary file 3 [file Table_3.docx]

**Table S3** Adjusted^a^ associations between MTL (T/S ratio) and PTA hearing thresholds stratified by race (N=2027)

| Race | N | Low-frequency PTA | Speech-frequency PTA | High-frequency PTA |
| --- | --- | --- | --- | --- |
|  |  | β (95% CI), *P* value of PTA levels, dB | | |
| Mexican American | 494 | -0.97 (-3.26, 1.32), 0.4055 | -0.72 (-3.18, 1.75), 0.5688 | 0.63 (-3.54, 4.81), 0.7660 |
| Non-Hispanic White | 986 | -1.02 (-2.62, 0.58), 0.2121 | -1.23 (-2.99, 0.54), 0.1731 | -1.94 (-5.25, 1.37), 0.2498 |
| Non-Hispanic Black | 344 | 0.14 (-2.42, 2.69), 0.9171 | -0.38 (-2.91, 2.14), 0.7675 | -1.47 (-5.15, 2.21), 0.4336 |
| Other races | 203 | -2.02 (-5.81, 1.76), 0.2956 | -2.17 (-6.06, 1.73), 0.2766 | 1.23 (-4.60, 7.06), 0.6797 |
| *P*_interaction_ |  | 0.3358 | 0.4834 | 0.1502 |

^a^ Adjusted for age, sex, education level, BMI, hypertension, diabetes, cigarette smoking, noise exposure.
